# Is Resistance Training an Option to Improve Functionality and Muscle Strength in Middle-Aged People with Multiple Sclerosis? A Systematic Review and Meta-Analysis

**DOI:** 10.3390/jcm13051378

**Published:** 2024-02-28

**Authors:** Javier Cano-Sánchez, Agustín Aibar-Almazán, Fidel Hita-Contreras, Diego Fernando Afanador-Restrepo, Antonio Martínez-Amat, Alexander Achalandabaso-Ochoa, María del Carmen Carcelén-Fraile

**Affiliations:** 1 Department of Health Sciences, Faculty of Health Sciences, University of Jaén, 23071 Jaén, Spainaaochoa@ujaen.es (A.A.-O.); 2 Faculty of Health Sciences and Sport, University Foundation of the Área Andina-Pereira, Pereira 660004, Colombia; 3 Department of Education and Psychology, Faculty of Social Sciences, University of Atlántico Medio, 35017 Las Palmas de Gran Canaria, Spain

**Keywords:** multiple sclerosis, demyelinating disease, resistance training, strength training, middle aged

## Abstract

**Background:** Currently, it is essential to adopt physical therapy strategies, such as resistance training, to enhance muscle strength and gait in middle-aged individuals (ages 45–65) suffering from Multiple Sclerosis. This is crucial in combating the typical symptoms of neurodegenerative diseases associated with functional loss. The objective of this study is to determine the effects of resistance training interventions on walking and muscle strength in middle-aged people with Multiple Sclerosis. **Methods:** A systematic review with meta-analysis was conducted by searching specific keywords in the PubMed, Scopus, Cochrane, and Web of Science databases. For inclusion, studies had to incorporate resistance training as a primary or significant component of the overall intervention for middle-aged patients with MS. Out of the 3675 articles identified, 12 randomized clinical trials met the criteria for inclusion in the review, with resistance training being a consistent feature in all of them. **Results:** Muscle strength and gait were evaluated as the main variables, with fatigue and the quality of life as secondary variables. This review reveals that resistance training significantly improves muscle strength. Resistance training achieves modest and non-significant improvements in gait. Notably, studies combining resistance training with motor control exercises achieve results of greater clinical significance in terms of gait. However, resistance training yields variable positive effects on perceived fatigue and the quality of life. **Conclusion:** Resistance training is useful for improving muscle strength; however, walking needs to be combined with motor control training.

## 1. Introduction

Multiple Sclerosis (MS) is a chronic neurodegenerative disease with great variability in terms of clinical course, levels of disability, signs, and symptoms [1]. It is characterized by the demyelination process of neuronal axons and the appearance of inflammatory plaques in the white matter [2,3]. Depending on the disease pattern, functional, psychological, and cognitive domains may be affected [4]. Additionally, the progressive nature of MS causes dysfunctions that diminish the quality of life [5].

The etiology of MS is multifactorial, involving genetic and environmental factors that contribute to the autoimmune process; however, a clear cause has not yet been established [6]. The primary risk factors for MS tend to include a family history, vitamin D deficiency resulting from low sun exposure, previous viral infections (especially Epstein–Barr virus), smoking, obesity, and female gender being among the most prominent [7]. MS is characterized by the occurrence of outbreaks or exacerbations, defined as the appearance or worsening of symptoms attributed to MS. These episodes present prolonged neurological changes that are more or less reversible and recur periodically based on the appearance or repetition of outbreaks, leading to neurological and functional sequelae [8]. It is estimated that 2.5 million people worldwide suffer from MS, with an increased incidence in southern parts of the world [9,10]. Regarding gender distribution, there exists a 2:1 ratio of women to men. Despite the peak onset age being 20 to 40 years, a slight increase in late-onset MS cases is being observed from the age of 50 onwards, regardless of gender [11,12].

Additionally, in the middle-aged population and older (age > 44), the level of frailty significantly increases and functions as a predictor of mortality, falls, worsening disability, hospitalization, and admission to residential centers. The primary indicators of frailty include decreased muscle strength, impaired functionality, weight loss, reduced physical activity, and increased fatigue [13]. Particularly in MS, symptoms associated with lack of physical activity such as spasticity, muscle weakness, fatigue, imbalance, and reduced physical fitness are prevalent, especially in middle-aged and older individuals [14]. To prevent, delay, and even achieve significant improvements in these symptoms, a proper prescription of physical exercise and progressive training adaptation is crucial. This can lead to an increase in overall physical fitness and improvements in psychological well-being [15]. Given the recognized benefits of exercise, a fundamental aspect in the treatment of these patients is the promotion of physical activity to counteract physical deconditioning and alleviate other symptoms. The ultimate goal is to potentially improve disability and enhance the overall quality of life [16].

At the musculoskeletal level, studies indicate that up to 70% of middle-aged MS patients show muscle weakness in one or more muscle groups, with a progressive decline in muscle strength throughout the disease [17,18]. Within physical exercise, resistance training emerges as a popular and effective modality to improve muscle function, functional performance, and health parameters in a broad spectrum of both healthy and clinical populations [19]. This training modality is typically characterized by a periodic and progressive approach with an emphasis on individualization. It involves working with two to three sets of one to two multi-joint exercises per major muscle group, aiming to achieve intensities ranging from 70% to 85% of the one-repetition maximum (1RM), and a frequency of two to three times per week, including concentric strength exercises performed at higher speeds and with moderate intensities (i.e., 40%–60% of 1RM) [20]. Among the anticipated outcomes of this training approach are increased muscle size and strength, contributing to improvements in performance, endurance, and overall health [21].

Closely associated with the reduction in muscle strength is the subsequent functional limitation experienced by MS patients, affecting aspects such as gait, quality and speed of movement, balance, and coordination. This leads to associated problems such as an increased risk of falls and morbidity [22,23]. Thus, apart from the potential improvements in the overall functionality of patients with resistance training, it may be interesting to combine it with other types of training that aim to increase motor control. Motor control training aims to restore the proper use of stabilizing muscles and reverse motor control deficits to enhance muscle activation, improve motor patterns, proprioception, and task execution [24]. This type of training generally focuses on exercises with special emphasis on correct biomechanical function and physiological joint position in space, producing positive results in tissue functional capacity, pain, and coordination [25]. This, in turn, helps reduce the risk of falls and improves balance, mobility, and postural hygiene [26].

It is known that the progressive advancement of the disease can lead to an increase in fatigue levels experienced by patients, which, along with other complications mentioned earlier, contributes to a decrease in the quality of life and the onset of mental health problems [27]. Studies support that resistance training is a suitable tool to mitigate fatigue, and the combination of this training modality with motor control training may be beneficial, promoting a subsequent increase in the patients’ quality of life [28].

In line with the aforementioned data and in the context of neurodegenerative diseases, scientific literature suggests that physical exercise is a widely used and safe treatment modality with positive outcomes in physical, cognitive, and psychological domains [29]. Furthermore, in the face of a public health issue such as MS, resistance training proves to be a cost-effective, easy-to-implement, and viable intervention method for this population [30]. Therefore, the aim of this systematic review is to analyze published data on the effectiveness of resistance training as a treatment method for MS patients, evaluating its impact from physical and functional perspectives, taking into account the influence of motor control work on gait and overall functionality, and how it can affect the quality of life.

## 2. Materials and Methods

This systematic review aims to assess the effectiveness of resistance training as a treatment for adult patients with MS. In conducting this review, we adhered to the protocols outlined in the 2020 PRISMA declaration [31] and followed the pre-specified protocol registered in PROSPERO (CRD42023462834). Additionally, we adhered to the methodological recommendations outlined in the “Cochrane Manual for the Elaboration of Systematic Reviews of Interventions” [32].

### 2.1. Sources of Information

The literature search was carried out between July and August 2023, utilizing the PubMed, Scopus, Cochrane, and Web of Science (WOS) databases.

### 2.2. Search Strategy

Several keywords were used in the search string, varying its format depending on the database used as follows:

PubMed: (multiple sclerosis [tiab] OR relapsing remitting multiple sclerosis [tiab] OR demyelinating disease [tiab]) AND (resistance training [tiab] OR strength training [tiab] OR muscle strength [tiab] OR muscle power [tiab] OR muscle weakness [tiab] OR muscle fatigue [tiab]).

Scopus: (“multiple sclerosis” OR “relapsing-remitting multiple sclerosis” OR “demyelinating disease”) AND (“resistance training” OR “strength training” OR “muscle strength” OR “muscle power”).

Cochrane and Web of Science: (multiple sclerosis OR relapsing remitting multiple sclerosis OR demyelinating disease) AND (resistance training OR strength training OR muscle strength OR muscle power).

### 2.3. Inclusion Criteria

The included articles were required to meet the following inclusion criteria: (i) randomized clinical trials (RCTs) as the article type, (ii) studies pertaining to resistance training in individuals diagnosed with MS, with the existence of at least one intervention group incorporating resistance training, (iii) studies centered on middle-aged and older individuals, specifically, where the mean age of participants was over 45 years, and (iv) articles published within the last 10 years.

### 2.4. Exclusion Criteria

We excluded studies which included the presence of other conditions such as cancer, cerebrovascular accident (CVA), cardiovascular (CVD), pulmonary, and/or renal disease. Additionally, studies were excluded if participants experienced acute flare-ups within the 6 months prior to the study, if they had participated in any exercise or rehabilitation programs within the 6 months preceding the study, or if the studies involved younger participants or individuals with a mean age of less than 45 years.

### 2.5. Study Selection Process

The initial screening involved the elimination of duplicate articles and those without available abstracts. Subsequently, titles and abstracts were thoroughly reviewed to exclude articles that did not align with the previously specified eligibility criteria. Finally, full-text articles underwent examination to confirm whether they met the inclusion criteria. The screening process was carried out independently by two authors (A.A.-A. and F.H.-C.). Any discrepancies were resolved through consensus with a third author (J.C.-S.). Data extraction encompassed various elements, including authors, year of publication, location, population details (sample size, age, and group distribution), study design, outcomes, measurement tools employed, description of intervention procedures, measurement time points, attrition rates, adverse effects, and main findings.

### 2.6. Data Extraction

The main variable evaluated in this study was muscle strength, while gait was assessed as a secondary variable considering functional domains such as balance, speed of movement, and aerobic endurance. Fatigue and the quality of life were also included as secondary variables.

### 2.7. Assessment of Methodological Quality

The methodological quality of the included studies was assessed using the PEDro scale [33]. This instrument comprises a checklist of 11 items, with a maximum score of 10 points, as the first item (“eligibility criteria”) is not factored into the final score. Each item is rated as either “Yes” (1 point) or “No” (0 points). Studies were categorized based on their scores: 0 to 3 points denoted “poor” quality, 4 to 5 points were considered “fair,” 6 to 8 points were categorized as “good,” and scores exceeding 9 points were regarded as “excellent” [34].

### 2.8. Analytic Decisions for Meta-Analysis

The results are presented in a forest plot, displaying the lead author, publication date, sample size, individual effects using the Hedge index (g), and the overall effect with a 95% confidence interval, along with the associated *p*-value. The use of the random effects model or the fixed effects model will be determined based on the heterogeneity and variability observed through Cochrane Q and I2.

For the meta-analysis, only those studies that maintained the usual care in the control group or were assigned to the waiting list were taken into account. For stratified or subgroup analysis, studies were grouped based on the type of intervention used and separated meta-analyses were performed within each group. This approach allowed for the assessment of variability and effect size within each subgroup, providing a more nuanced understanding of the results. Finally, the risk of publication bias was assessed using a funnel plot.

## 3. Results

**Figure 1:**
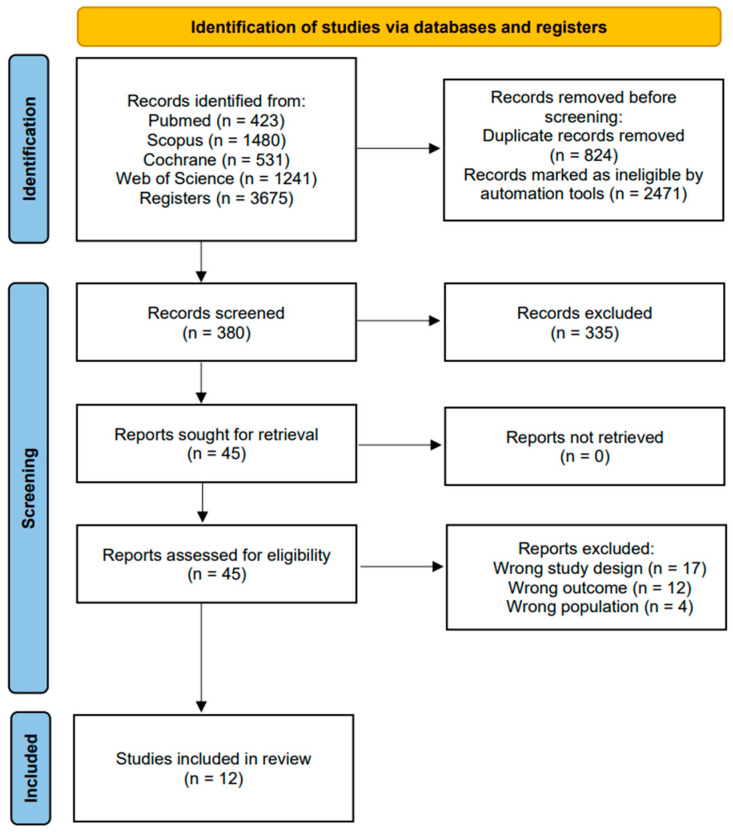
Flowchart of the study selection process.

The initial search across different databases yielded a total of 3675 articles. Subsequently, in the same databases, the search was refined by document type (article and randomized clinical trial), year of publication (last 10 years), and participant age (Middle Age: 45–64 years, Advanced Age: >65 years, Middle Age + Advanced Age: >45 years), followed by the removal of duplicate articles. This process resulted in 380 unique articles. The 380 articles underwent title and abstract evaluation, and 45 articles emerged as candidates for qualitative evaluation. Ultimately, 12 final articles [35,36,37,38,39,40,41,42,43,44,45,46] were selected for the meta-analysis, as they met the inclusion criteria, while 33 articles were excluded (see Figure 1).

### 3.1. Methodological Quality

The methodological quality of the included studies was assessed using the PEDro scale. The scores of 11 studies [36,37,38,39,40,41,42,43,44,45,46] were obtained from the PEDro web portal, while the rest were assessed manually [35]. Among the included studies, 9 were rated as Good, while only 3 were rated as Fair [35,37,44]. It is important to highlight that none of the studies blinded participants or therapists (Table 1).

### 3.2. Characteristics of the Studies

All the articles included in this systematic review with meta-analysis were randomized controlled clinical trials, conducted in various countries, including Norway [36,45], Denmark [39], Italy [40,43,46], Ireland [41], Canada [35], the United States [37,38,42], and Spain [44]. A total of 459 individuals participated in the studies included in this review, with 207 assigned to the control group and 252 receiving a resistance exercise intervention. The mean age of the participants was 49.69 ± 9.26 years. Regarding the control groups, six of the articles [38,39,40,41,44,46] compared the intervention with usual care or wait list, while the rest compared it to a different intervention (Table 2).

### 3.3. Outcomes

The current systematic review with meta-analysis has muscle strength as the primary variable, with gait, fatigue, and the quality of life as secondary variables. Muscle strength was evaluated in six of the clinical trials, measured through isometric maximum voluntary contraction, leg press strength, or a dynamometer, targeting various muscle groups in the lower limbs (MMII). Among the included studies, eight assessed gait parameters using tests such as the 2-Minute Walking Test (2MWT), the 6-Minute Walking Test (6MWT), Timed 25-Foot Walk (T25FW), Functional Ambulation Profile (FAP), or the Multiple Sclerosis Walking Scale (MSWS-12). Additionally, fatigue was examined in six of the included studies using the Modified Fatigue Impact Scale (MFIS), Fatigue Severity Scale (FFS), and Fatigue Index (FI) as measurement instruments; the quality of life was assessed with Multiple Sclerosis Quality of Life-54 (MSQOL-54), Multiple Sclerosis Impact Scale (MSIS), and Leeds MS Quality of Life Scale (LMSQOL).

#### 3.3.1. Muscle Strength

Among the six included studies that assessed the impact of resistance exercises on muscle strength in patients with multiple sclerosis, five reported statistically significant improvements, while one study [41] indicated no significant changes (*p* > 0.05). Correale et al. [40] evaluated the effects on lower limb extension and observed improvements assessed at 1RM (*p* < 0.05). Callesen et al. [39] and Medina-Perez et al. [44] evaluated the effects on knee movements; Callesen et al. observed a mean difference in the isometric maximum voluntary contraction produced in knee extension of 0.17 [−0.02; 0.36], *p* = 0.08, while Medina-Perez et al. reported statistically significant intra- and inter-group changes in different measurement points, favoring the control group (baseline: 754 ± 235; post-intervention: 811 ± 283; Follow-up: 755 ± 234, *p* < 0.05), indicating a significant post-intervention decrease. Akbar et al. [35] and Manca et al. [43] reported favorable intervention effects using hand (d = 1.11) and ankle (F = 4.02; *p* = 0.01) dynamometry, respectively.

**Figure 2:**
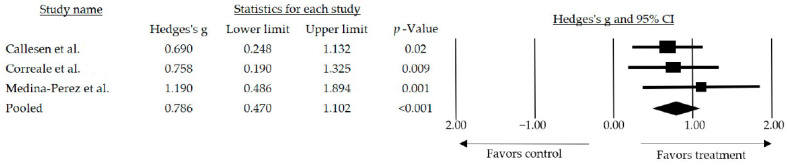
Forest plot of the overall effect of resistance training and resistance training combined over muscle strength outcomes. The black box represents the point estimate for the respective study, while the size of the box represents the population size, and the horizontal line is the 95% CI. The diamond-shaped figure represents the estimated point of the mean effect size [39,40,44].

In the meta-analysis, the decision was made to use the random effects model (I^2^ = 0%; Q-Value = 0.909 with 2 degrees of freedom). Additionally, we prioritized the use of the 423 random effects model to extrapolate the results obtained through the meta-analysis. The mean effect size observed among the studies that included any strength-related variables and compared to a usual care group was medium and statistically significant (*g* = 0.786, 95% CI: 0.470–1.102; *p* < 0.001) (Figure 2).

#### 3.3.2. Walk Performance

**Figure 3:**
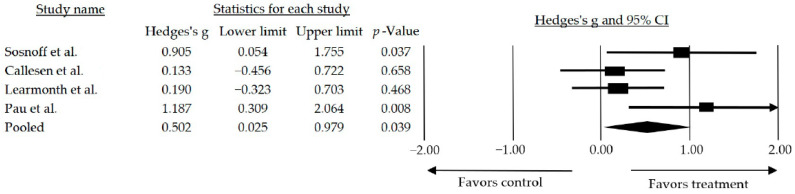
Forest plot of walk performance for resistance training alone or in combination compared to a no treatment/wait list control. The black box represents the point estimate for the respective study, while the size of the box represents the population size and the horizontal line is the 95% CI. The diamond-shaped figure represents the estimated point of the mean effect size. The arrow represents that the point estimate for this study is more than 2.00 [38,39,42,46].

Out of the 12 articles included, 8 assessed gait of which 4 [36,37,38,46] showed statistically significant improvements. However, only four were included in the meta-analysis since only those compared the intervention with a waiting list or usual care. The remaining studies compared the intervention with usual care combined with walking [36], stretching and toning activities [37], resistance training [41], and treadmill training [45]. Two models were considered for this meta-analysis: the random and the fixed. However, the random model was chosen (I^2^ = 49%, Q-Value = 5.986 with 3 degrees of freedom, and *p* = 0.112). The mean effect size observed when comparing studies that used interventions based on resistance training alone and those combined with other training was medium and statistically significant (*g* = 0.502, 95% CI: 0.025–0.979; *p* = 0.039) (Figure 3).

**Figure 4:**
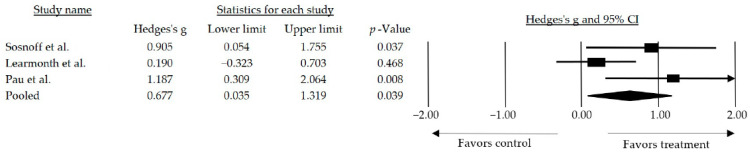
Forest plot of the overall effect of resistance training combined over walk performance. The black box represents the point estimate for the respective study, while the size of the box represents the population size and the horizontal line is the 95% CI. The diamond-shaped figure represents the estimated point of the mean effect size. The arrow represents that the point estimate for this study is more than 2.00 [38,42,46].

Furthermore, a subgroup analysis was conducted by categorizing the studies based on the type of intervention performed. The effect size observed when the studies combined resistance exercise with another type of training requiring some degree of motor control was medium and statistically significant (*g* = 0.677, 95% CI: 0.035–1.319; *p* = 0.039) (Figure 4).

#### 3.3.3. Fatigue

Six out of the twelve studies included in the systematic review assess the level of fatigue in patients with MS, however, only three studies [39,40,42] employed a consistent control group (usual care or waitlist). Of all the articles studying fatigue, only one found significant improvements in fatigue in the group × time interaction [35]. Two studies observed differences between groups favoring the intervention group [39,40], and one attributed the effects to the time interaction [43]. In contrast, the remaining interventions did not yield significant improvements in terms of fatigue.

Furthermore, a meta-analysis was conducted including the studies where the control group received no treatment or was on the waiting list [39,40,42]. The randomized model was employed for this meta-analysis (I2 = 19%, Q-Value = 6.983 with 2 degrees of freedom, and *p* = 0.030). However, the observed mean effect was small and lacked statistical significance (*g* = 0.505, 95% CI: −0.146–1.156; *p* = 0.128).

#### 3.3.4. The Quality of Life

Two out of the total selected articles assessed the quality of life. In the first one [40], the results show significant improvements when comparing both groups; however, in the second one [42], improvements are obtained but do not reach statistical significance. In both studies, resistance training was compared to a no treatment/wait list control.

### 3.4. Adverse Events

Among the 12 studies incorporated into this systematic review with meta-analysis, only 5 studies were attentive to adverse events. Among these, four studies [38,41,44,45] reported no adverse events, while Callesen et al. [39] documented two adverse events within the resistance training group. Notably, one of these incidents resulted in a participant discontinuing the study due to extreme fatigue. Finally, the remaining studies did not address the presence or absence of adverse events during their respective randomized controlled clinical trials.

### 3.5. Publication Bias

Following the graphical analysis of the funnel plot, it was possible to rule out a potential publication bias due to the symmetry evidenced in the distribution of the graph.

## 4. Discussion

This systematic review with meta-analysis encompasses 12 randomized controlled clinical trials [35,36,37,38,39,40,41,42,43,44,45,46] that investigated the impact of resistance training on middle-aged patients with multiple sclerosis (MS), specifically those aged 45 years and older. The main objective of the review was to assess the impact of resistance training on muscle strength, with additional attention given to factors such as gait, fatigue, and the quality of life. The results obtained from the meta-analysis suggest a significant advantage in favor of interventions based on resistance training. These findings underscore the potential benefits of incorporating resistance-based training for middle-aged adults diagnosed with MS, highlighting its positive impact on overall health and the quality of life.

In terms of methodological quality, the majority of the included articles, specifically nine [36,38,39,40,41,42,43,45,46], demonstrated good methodological quality with a low risk of bias. Only three studies [35,37,44] were rated as medium quality studies according to the PEDro methodological scale. It is worth noting that none of the selected articles achieved the maximum score for excellence. The absence of blinding for therapists and participants is a notable aspect. These deficiencies, observed frequently in the selected articles, have the potential to influence the observed results. Various studies report the potential of up to a 7% increase in result exaggeration due to the lack of blinding in participants and therapists [47].

In individuals affected by MS, the augmentation or preservation of muscle strength holds particular importance in addressing various degenerative processes, potentially alleviating their impact and thereby enhancing the quality of life for these individuals. Physical deconditioning is a prevalent issue in this population [48]. The analysis conducted in this review supports the viability of interventions based on resistance training, revealing a medium and significant mean effect size. Importantly, the pooled effect size observed included participants with different levels of disability, assessed with the Expanded Disability Status Scale (EDSS) scores. Among the six studies used to measure the effect of resistance exercises on people with multiple sclerosis, the meta-analysis reflects a medium effect size that is statistically significant between studies that included any variable related to muscle strength compared to a usual care group (*g* = 0.786, 95% CI: 0.470–1.102; *p* < 0.001). These findings underscore the potential of resistance training interventions, indicating their possible effectiveness in improving muscle strength in individuals with MS.

Other previous studies [49,50,51] concur that resistance training could be a valuable tool for individuals with MS who experience compromised functional capacity, challenges in performing the activities of daily living, or heightened levels of fatigue—a notable indicator of muscle function and applicable to the perceived level of frailty. However, another previous review [52] suggests that consistent improvements in muscle strength do not always translate into enhancements in balance, functional capacity, and fatigue. This variability is thought to stem from the adaptation of strength specific to the tasks addressed during the intervention. Dalgas et al. [53], for example, found that after 12 weeks of resistance training focused on the lower limbs, they did not observe improvements in hand grip strength. This underscores the need to perform resistance training with the muscles required for the functional activities of interest, selecting the most appropriate method for evaluating and measuring changes. The choice of specific tasks targeted during the intervention can significantly influence the outcomes and their generalization to broader aspects of physical function.

Contrary to the positive results observed in the previously discussed studies, the article by Uszynski et al. [41] did not yield significant results in terms of muscle strength improvement. This could be attributed to the unique design of their study, where they compared two resistance exercise interventions—one involving resistance exercise alone and the other combining body vibration with resistance exercise. It is important to note that in other studies [35,43,54,55], when interventions involving resistance training were compared with controls without intervention or with multidisciplinary care, significant improvements were reported. This underscores the influence of specific intervention components and highlights the need for careful consideration of the intervention design and comparison groups in interpreting study outcomes.

Concerning gait, the progressive nature of the disease and its neurodegenerative consequences can lead to alterations in the physiological gait pattern, changes in rhythm and mechanics, and associated motor control alterations, including loss of balance, increased fatigue, and an elevated risk of falling. These factors collectively impact the quality of life and contribute to increased frailty in individuals with MS [56]. Of the 12 articles included in the systematic review with meta-analysis, 8 [36,37,38,39,41,42,45,46] examined gait. The analysis revealed a medium and statistically significant effect size when resistance training was used as an intervention on its own or in combination with other types of training (*g* = 0.502, 95% CI: 0.025–0.979; *p* = 0.039) compared to a no treatment/wait list control group. Subsequently, a subgroup analysis was conducted by categorizing the studies based on the type of intervention performed, showing that the inclusion of resistance-based interventions in conjunction with some form of motor control resulted in a larger effect size observed (*g* = 0.677, 95% CI: 0.035–1.319; *p* = 0.039) compared to the effect size observed when all studies were included (*g* = 0.502, 95% CI: 0.025–0.979; *p* = 0.039).

The modest and non-significant benefits on gait observed in studies using only resistance training may be attributed to variations in equipment and workplaces, potentially impacting the level of disability measured by the Expanded Disability Status Scale (EDSS) [57]. In contrast, when resistance training is combined with other interventions such as motor control training, particularly focusing on balance and proprioceptive work, it may lead to greater improvements. This broader focus is less specific in strengthening a particular muscle group. Certain studies [58,59] have highlighted a stronger association between balance and gait speed than between balance and muscle strength in MS patients. This suggests that alterations in balance control might contribute more to the metabolic cost of gait, potentially impacting gait speed. 

Regarding fatigue and its influence on the frailty of the population under consideration, previous studies have demonstrated the efficacy of resistance exercise as a mitigating factor for this effect [60]. Additionally, noteworthy is the impact that the combination of resistance training with motor control training can have on fatigue, leading to significant improvements in fatigue levels [61]. Considering the variability observed in fatigue, despite achieving positive results, statistical significance is not reached in all cases. The fact that some studies show improvements in both groups without finding significant changes between groups may be due to strict compliance levels and required activity. This aspect was also evident in the meta-analysis conducted within the scope of this review (*g* = 0.505, 95% CI: −0.146–1.156; *p* = 0.128).

Following this, the physical and cognitive conditions resulting from MS can, in turn, influence the quality of life and mental health of affected individuals. 

The findings derived from the systematic review with meta-analysis reveal some positive effects of resistance training on the quality of life of individuals with MS. However, it is imperative to note that this assertion regarding the quality of life outcomes is based on two studies [40,42], with statistical significance observed only in one [40]. Multiple studies support improvements in various domains, including energy, emotional well-being, health problems, and mental and physical subscales [62,63]. Moreover, similar benefits have been observed in other neurological populations, such as Parkinson’s disease, Amyotrophic Lateral Sclerosis (ALS), and patients convalescing after stroke [64,65,66].

Finally, compiling the above and considering which aspects of the evaluated interventions in the selected articles may lead to greater improvement in muscle strength and functional mobility, we consider two possible reasons. The first involves the coincidence that improvements in muscular strength come from progressive resistance training interventions [35,44], characterized, as seen in some previous research [67], by the constant adaptation of the load, the number of sets, repetitions, or the increase in difficulty in the execution of exercises, as well as by focusing on specific muscle work. This progression in training could help muscular adaptation by increasing strength and muscular endurance, thereby contributing to mitigating fatigue levels [36,37,46]. The second reason arises because more substantial improvements in functional outcomes occur when resistance training is combined with motor control training, responding more to a global biomechanical approach that places greater importance on a set of fundamental aspects to achieve optimal gait than solely on the pursuit of hypertrophy and muscle strength gain. Overall, the importance of a well-designed training program must be added, noting that when exercise selection is appropriate and aligns with specific goals for each task, there is a greater possibility of achieving the overall objectives outlined by combining different training modalities.

This systematic review with meta-analysis has several limitations that should be acknowledged. It is necessary to mention the scarcity of studies and the small sample sizes, leading to a lack of statistical power. Another notable limitation is the lack of blinding, both for therapists responsible for treatment delivery and for the participants. This absence of blinding may introduce bias and compromise the objectivity of the results. Another significant limitation pertains to adverse events, as only a few studies have reported them, thereby hindering the ability to ascertain the safety profile of the intervention. Additionally, there was great diversity in the intervention protocols tested in the different studies, as well as in the comparison conditions. There is also geographical diversity among the studies included in the review, which may pose a challenge for generalizing the results, as the majority of the studies were conducted in Europe, with the remainder in North America. Therefore, caution should be exercised when applying these findings to broader populations or other geographical regions.

## 5. Conclusions

This systematic review with meta-analysis, comprising 12 RCTs, indicates that a resistance training intervention for middle-aged individuals with multiple sclerosis leads to significant improvements in terms of muscle strength and variable improvements in perceived fatigue and the quality of life, with improvements in perceived fatigue lacking statistical significance. However, the benefits of resistance training did not extend to gait, producing modest and non-significant improvements compared to control groups. It is noteworthy that studies combining resistance training with motor control exercises achieve results of greater clinical significance in terms of gait. These findings underscore the potential of resistance training to prevent and ameliorate symptoms of physical functional impairment, offering an opportunity for early intervention to mitigate the impact of MS-related conditions. Healthcare professionals should consider the ongoing use of resistance training-based interventions for this demographic, given the favorable cost–benefit balance. However, to fortify the current evidence base, there is an urgent need for more research efforts in this area, employing larger sample sizes and employing higher-quality randomized methods. Additionally, further research is required to determine the best protocols and patient subgroups that will benefit most from this well-known and easily implemented intervention.

## Figures and Tables

**Table 1:** Methodological quality of the included articles.

	1*	2	3	4	5	6	7	8	9	10	11	Total Score
Akbar et al. [35]	Y	Y	N	Y	N	N	N	Y	Y	Y	Y	5
Arntzen et al. [36]	Y	Y	Y	Y	N	N	Y	Y	N	Y	Y	7
Sandroff et al. [37]	Y	Y	N	Y	N	N	N	N	Y	Y	Y	5
Sosnoff et al. [38]	Y	Y	Y	Y	N	N	Y	N	N	Y	Y	6
Callesen et al. [39]	Y	Y	Y	Y	N	N	Y	Y	N	Y	Y	7
Correale et al. [40]	N	Y	N	Y	N	N	Y	Y	N	Y	Y	6
Uszynski et al. [41]	Y	Y	Y	Y	N	N	Y	Y	N	Y	Y	7
Learmonth et al. [42]	Y	Y	Y	Y	N	N	N	Y	N	Y	Y	6
Manca et al. [43]	N	Y	Y	Y	N	N	Y	Y	N	Y	Y	7
Medina-Perez et al. [44]	Y	Y	N	Y	N	N	N	Y	N	Y	Y	5
Braendvik et al. [45]	Y	Y	N	Y	N	N	N	Y	Y	Y	Y	6
Pau et al. [46]	Y	Y	N	Y	N	N	N	Y	Y	Y	Y	6

Items: 1* = eligibility criteria (it is not taken into account for the final score); 2 = random allocation; 3 = concealed allocation; 4 = baseline comparability; 5 = blind subjects; 6 = blind therapists; 7 = blind assessors; 8 = adequate follow-up; 9 = intention-to-treat analysis; 10 = between group comparisons; 11 = point estimates and variability; Y = yes; N = no.

**Table 2:** Characteristics of the included studies.

Author and Year	Sex	Sample CG/IG		Control Group	Intervention Group				Results
			Disability Range of the Included Patients		Age Mean ± SD Median (IQR)	Treatment	Exercise Parameters	EDSS Score Pre-Intervention Mean ± SD Median (IQR)	
Akbar et al. [35]	F: 100%	5/5	Fully ambulatory/ability to walk without an assistive device	Stretching	45.6 ± 12.8	Progressive resistance training	I: Adjusted to each participant F: 3 times/week #S: 48 sessions D: 600 min	Not reported	Progressive resistance training improves fatigue (main effect of time: F = 0.84, d = 0.65), functional connectivity between the left inferior caudate and parietal (F = 66.0, *p* < 0.001), bilateral frontal (both *p* < 0.001), and right insula (F = 21.8, *p* = 0.002) regions and grip strength (d = 1.11).
Arntzen et al. [36]	F: 69.2% M: 30.8%	40/39	EDSS 1–6.5; mean = 2.37	Usual care + walking	52.2 ± 12.9	Dynamic core stability training	I: Adjusted to each participant F: 3 times/week #S: 18 sessions D: 60 min	2.45 ± 1.65	Dynamic core stability training significantly improved walking (2MWT) immediately after the intervention for up to 24 weeks of follow-up (Post: 6.7 m, 95% CI [8.15, 25.25], *p* < 0.001; Follow-Up: 15.08 m, 95% CI [6.39, 23.77] *p* = 0.001).
Sandroff et al. [37]	F: 85.5% M: 14.5%	40/43	EDSS 4–6; PDDS mean = 3.5	Stretching and toning activities	49.8 ± 8.5	Aerobic, resistance, and balance exercise	I: Vigorous F: 3 times/week #S: 18 sessions D: 25 min	Not reported	This RCT provides novel, preliminary evidence that multimodal exercise training may improve endurance walking (*r* = 0.25) performance and cognitive processing speed.
Sosnoff et al. [38]	F: 77.7% M: 22.3%	14/13	EDSS 2.5–6.5; mean = 5	Waiting list	60.0 ± 6.1	Balance, walking and resistance training	I: Adjusted to each participant F: 3 times/week #S: 38 sessions D: 45–60 min	5.5 ± 2.5	A home-based exercise program enhanced walking (T25FW, Pre: 6.6 ± 1.3; Post: 6.4 ± 1.4; *p* = 0.040).
Callesen et al. [39]	M: 23% F: 77%	20/23	EDDS 2.0–6.5; mean = 3.5	Usual care	52 (30–75)	Progressive resistance training	I: Moderate F: 2 times/week #S: 21 sessions D: 60 min	4 (2–6.5)	Progressive resistance training reduced fatigue impact, however, had no impact on gait when compared to control group (Mean diff: 0.02; 95% CI [−0.08; 0.13], *p* = 0.660)
Correale et al. [40]	F: 100%	9/14	Mean EDSS = 2.25	Usual care	45.4 ±7.2	Endurance and resistance training	I: Moderate to Vigorous F: 2 times/week #S: 24 sessions D: 30 min	Not reported	Endurance and resistance training leads to enhanced muscle strength, along with decreased fatigue, depressive symptoms, and greater overall health-related quality of life (*p* < 0.05). Notably, these positive changes endure even after a 12-week period of detraining.
Uszynski et al. [41]	M: 28.57% F: 71.43%	13/14	Participants with MS who walked independently or used an assistive device with scores of 0, 1, 2, and 3, inclusive on the Guys Neurological Disability scale (GNDS).	Resistance training	45.5 (38.5–52.3)	Vibration + resistance training	I: Moderate to Vigorous F: 3 times/week #S: 36 sessions D: 20 min	Not reported	No between group differences were found for muscle strength, balance, or gait (*p* > 0.05).
Learmonth et al. [42]	F: 96.55% M: 3.45%	28/29	EDSS 1–6; mean = 1.5	Waitlist	48.4 ± 9.7	Resistance and aerobic training	I: Mild to moderate F: 4 times/week #S: 64 sessions D: 45 min	1.25 ± 2.5	A small, non-statistically significant effect size of combined exercise on MSWS-12 in patients with MS is presented. (Cohen’s D: −0.10, *F:* 0.47)
Manca et al. [43]	F: 80% M: 20%	15/15	EDDS ≤ 6; mean = 3.4	Contralateral resistance training	47.3 ± 9.4	Direct resistance training	I: Vigorous F: 3 times/week #S: 18 sessions D: 25 min	3.0 ± 1.00	Both direct and indirect resistance training led to significant gains in muscle strength. However, only direct resistance training increased walking speed (Pre: 085 ± 0.14; Post: 0.99 ± 0.15; *p* < 0.0001)
Medina-Perez et al. [44]	NR	12/30	Mean EDSS = 4.3	Usual care	49.6 ± 11	Resistance training	I: Vigorous F: 3 times/week #S: 18 sessions D: 25 min	4.5 ± 2.1	A 12-week RTP improved extension, maximal voluntary isometric contraction, and muscle power in MS patients.
Braendvik et al. [45]	M: 34.6% F: 65.4%	11/15	EDSS ≤ 6; mean = 3.15	Treadmill training	49.1 ± 6.2	Resistance training	I: Moderate to Vigorous F: 3 times/week #S: 24 sessions D: 30 min	3.2 ± 1.4	Resistance training had no significant effect over gait assessed with the Functional Ambulation Profile (Pre: 91.7, Post: 90.3; *p* = 0.844)
Pau et al. [46]	M: 54.54% F: 45.45%	11/11	EDSS 1.5–5.5; mean = 3.5	Usual care	47.4 ± 10.8	Aerobic and resistance training	I: Moderate F: 3 times/week #S: 72 sessions D: 60 min	3.6 ± 0.9	Although some improvements have been observed, the substantial constancy of kinematic patterns of gait suggests that the full transferability of the administered training on the ambulation function may require more specific exercises.

Abbreviations. I: Intensity; F: Frequency; #S: Number of Sessions; D: Duration; CG: Control Group; IG: Intervention Group; SD: Standard Deviation; IQR: Interquartile Range; MS: Multiple Sclerosis; 2MWT: 2-Minute Walk Test; MSWS-12: 12-item Multiple Sclerosis Walking Scale; RTP: Resistance Training Program; RCT: Randomized Controlled Trial; T25FW: Timed 25-Foot Walk; GNDS: Guys Neurological Disability Scale; EDSS: Expanded Disability Status Scale.

## Data Availability

Not applicable.
